# Transgenerational plasticity following a dual pathogen and stress challenge in fruit flies

**DOI:** 10.1186/s12862-016-0737-6

**Published:** 2016-08-27

**Authors:** M. Nystrand, E. J. Cassidy, D. K. Dowling

**Affiliations:** School of Biological Sciences, Monash University, Clayton, VIC 3800 Australia

**Keywords:** Anticipatory effects, Maternal effects, Maternal stress, Parental effects, Phenotypic plasticity, Transgenerational effects, Transgenerational plasticity

## Abstract

**Background:**

Phenotypic plasticity operates across generations, when the parental environment affects phenotypic expression in the offspring. Recent studies in invertebrates have reported transgenerational plasticity in phenotypic responses of offspring when the mothers had been previously exposed to either live or heat-killed pathogens. Understanding whether this plasticity is adaptive requires a factorial design in which both mothers and their offspring are subjected to either the pathogen challenge or a control, in experimentally matched and mismatched combinations. Most prior studies exploring the capacity for pathogen-mediated transgenerational plasticity have, however, failed to adopt such a design. Furthermore, it is currently poorly understood whether the magnitude or direction of pathogen-mediated transgenerational responses will be sensitive to environmental heterogeneity. Here, we explored the transgenerational consequences of a dual pathogen and stress challenge administered in the maternal generation in the fruit fly, *Drosophila melanogaster*. Prospective mothers were assigned to a non-infectious pathogen treatment consisting of an injection with heat-killed bacteria or a procedural control, and a stress treatment consisting of sleep deprivation or control. Their daughters and sons were similarly assigned to the same pathogen treatment, prior to measurement of their reproductive success.

**Results:**

We observed transgenerational interactions involving pathogen treatments of mothers and their offspring, on the reproductive success of daughters but not sons. These interactions were unaffected by sleep deprivation.

**Conclusions:**

The direction of the transgenerational effects was not consistent with that predicted under a scenario of adaptive transgenerational plasticity. Instead, they were indicative of expectations based on terminal investment.

**Electronic supplementary material:**

The online version of this article (doi:10.1186/s12862-016-0737-6) contains supplementary material, which is available to authorized users.

## Background

Maternal effects occur when the maternal phenotype, or the environment in which the mother resides, shapes the expression of the offspring phenotype [1–3]. Hence, maternal effects can be traced to a diverse range of factors, from variation in maternal behavior to physiology, and can result in the differential transfer of nutrients, hormones, antibodies or epigenetic markers to offspring [1, 4–6].

Because of classic parent-offspring conflict over resources, maternal effects can have a negative influence on offspring fitness [3]. However, maternal effects can also be adaptive if they act to directly augment offspring fitness [1, 3, 7]. Such ‘positive’ maternal effects provide examples of adaptive transgenerational plasticity, sometimes referred to as ‘anticipatory maternal effects’ [1, 3, 8]. In particular, maternal effects are adaptive when an environmental challenge experienced by a mother prior to reproduction confers a relative increase in fitness to her subsequent offspring when they are themselves faced with a similar environmental challenge to that previously faced by their mother [3, 8]. As such, conclusive evidence for adaptive transgenerational plasticity can only be provided by studies that not only experimentally manipulate parental environments pre-reproduction, but also subsequently manipulate environments of the offspring, thereby either matching or mis-matching maternal and offspring environmental conditions [3, 8]. Such designs have successfully been adopted in a range of taxa; a classic example includes a study of seed beetles (*Stator limbatus*), in which eggs that were deposited in the same environment as that experienced by the mother had greater survival than eggs that were deposited in a novel environment [9]. Likewise, in a study of toxicant-resistance in bryozoans (*Bugula neritina*), offspring that were exposed to the same pollutant as their mothers had higher survival than offspring that were not [10], and a study of American bellflowers (*Campanulastrum americanum*), showed that offspring had higher fitness when their light environment was matched to that experienced by their mothers [11]. However, there are also a range of studies that have failed to find evidence for adaptive transgenerational plasticity [3, 12–14], and indeed, a recent meta-analysis concluded that there was only weak evidence for adaptive transgenerational plasticity across natural populations of plants and animals [8].

Numerous studies conducted in invertebrates have shown that pathogen challenges administered to females prior to reproduction can influence phenotypic expression in their offspring. In several cases, application of a maternal pathogen challenge has been associated with increased robustness of the offspring phenotype, including increased survival [15–17], reproductive success [18, 19] or augmented immune responses e.g. [15, 16, 20–22]. Because such effects have been shown to augment proxies of offspring fitness, it has been commonly inferred that they provide an adaptive benefit to offspring. Furthermore, because several studies have shown that such maternal pathogen challenges can affect the expression of offspring immune components (e.g. offspring phenoloxidase [PO] or antimicrobial activity), researchers have commonly concluded that such findings represent cases of ‘transgenerational immune priming’, and such priming effects have been suggested to be functionally similar to the transfer of acquired immunity in vertebrates (e.g. the actual transfer of antibodies) [4, 18, 23].

Yet, most of the studies exploring pathogen-mediated transgenerational effects in invertebrates suffer from two limitations. First, while there is evidence that mothers can enhance offspring immune function, there is no evidence that mothers transmit actual immune factors [24] in a manner similar to that of vertebrate adaptive immune priming (e.g. antibody transfer) [4, 5, 25]. Hence, any analogues between invertebrate ‘immune priming’ and the vertebrate adaptive immune system are contentious and in need of further research to specifically address the underlying mechanisms. This is because pathogen-induced transgenerational effects on immune expression could simply be a result of general condition-dependence, or be mediated by epigenetic changes to the DNA [1, 4, 24, 26–28]. Alternatively, maternally induced offspring immune augmentation may be caused by mothers transferring the pathogen *per se* (or parts thereof), hence triggering the offspring’s own immune system, as suggested in some recent studies that have demonstrated maternal transfer of bacterial fragments to eggs [24, 29].

However, the lack of precise mechanistic explanations is a relatively minor problem from an evolutionary standpoint. Of broader significance is the second limitation, which relates to the experimental design; most prior studies have failed to implement designs with the experimental power to detect whether transgenerational patterns were adaptive. It is for this exact reason that a recent meta-analysis surveying the evidence and effect sizes associated with adaptive trans-generational immunity, chose not to incorporate studies examining transgenerational consequences of pathogen-mediated challenges as part of their base of evidence [8]. We elaborate below.

The adaptive value of transgenerational immune effects, to both mother and offspring, will inevitably depend on the benefits that such effects bring to offspring produced by pathogen-exposed mothers relative to the costs imposed on the mother from investing in offspring immune-protection − a cost-benefit equation that could, for example, be heavily shaped by maternal condition-dependence if pathogen-challenged mothers are in poorer condition [30]. A prediction based on life history theory is that offspring of pathogen-treated mothers would exhibit higher relative fitness if they themselves are exposed to the same pathogen in their lifetime, but lower relative fitness otherwise (due to the costs associated with the pathogen challenge in the maternal generation). That is, from a life-history perspective, assessing the benefits of a maternal pathogen challenge across matched and mis-matched contexts is key to interpretation. Yet, only a handful of previous invertebrate studies have utilized experimental and analytical approaches that can fully dissect the adaptive nature of pathogen-mediated transgenerational plasticity on offspring fitness [15, 16, 18, 19, 31, 32], by harnessing factorial designs in which maternal and offspring immune environments are in fact matched or mis-matched [3, 8].

Furthermore, while it is well established that the magnitude and direction of maternal effects can be substantially altered depending on internal (e.g. maternal age, size) or external (e.g. environmental condition, diet) environment, e.g. [10, 33, 34], studies addressing such context-dependence in relation to pathogen-mediated maternal effects are limited, and most research comes from subjects that are assayed under benign and largely stress-free laboratory environments [35–37]. It thus remains unclear whether cases of adaptive pathogen-mediated transgenerational plasticity will be upheld when mothers are faced with multifaceted stresses similar to those likely to be experienced under natural conditions [35, 37].

Here, we set out to test whether interactions between a pathogen-challenge combined with a stress challenge in the maternal generation interact with a pathogen challenge in the offspring generation*,* to shape offspring reproductive success in fruit flies (*Drosophila melanogaster*). The pathogen challenge was a non-infectious challenge, in that the bacteria used (*Escherichia coli* mixed with *Micrococcus luteus*) had been heat-killed prior to use, thus activating maternal immune genes but without causing direct pathogenesis [19, 38]. We limited our assay to measurement of one key life-history trait in the offspring ─ reproductive success. This is a trait that has previously been shown to be sensitive to a similar heat-killed pathogen challenge in this species [19, 39]. We used sleep deprivation as a measure of maternal stress, because it is well-studied in *Drosophila.* Variation in sleep has been shown to interact with a number of physiological and behavioural traits in *Drosophila*, such immune function, metabolism, learning and memory function [40–48]. Moreover, sleep patterns are known to influence the expression of a number of immune-related genes [41, 44, 49, 50]. Of key interest to our study is prior evidence suggesting that flies experiencing sleep deprivation exhibit greater resistance to bacterial infection relative to controls [41, 51], suggesting that sleep-stress might play an important, but hitherto unexplored role in augmenting the strength of pathogen-mediated adaptive transgenerational effects.

The experiment was designed to test two main predictions. The first prediction was that interactions between maternal and offspring pathogen challenges on offspring reproductive success would conform to a pattern expected under adaptive transgenerational plasticity, with matched combinations of maternal and offspring pathogen exposure outperforming mis-matched combinations (i.e. pathogen-challenged offspring would perform better if their mothers had also been pathogen-challenged). The second prediction revolved around whether maternal sleep deprivation prior to reproduction would act to augment signatures of pathogen-mediated adaptive transgenerational effects or not, in female and male offspring.

## Methods

### Fly population

The flies used in this study originated from a natural population collected from three different localities in Coffs Harbour (New South Wales, Australia), in February 2010 [52]. Each of a total of 60 wild-caught, non-virgin females, contributed 10 sons and 10 daughters to form a mass-bred laboratory population, which was then maintained in pairs of 20 adults across twelve 10-dram vials, under standardized rearing conditions of 12 L: 12 D cycle, 25 °C, 6 ml of potato-dextrose-agar-yeast substrate and *ad libitum* access to live yeast per vial. The population was propagated using flies that were allowed a 20 h period to oviposit when 4 days of adult age. The eggs of each vial were then trimmed to between 80 and 100 per vial. Upon their eclosion into adulthood, offspring were admixed with offspring from all other vials prior to their separation and transfer into 12 new vials, each comprised of 20 pairs of adults.

At the onset of the experiment, the laboratory population had been maintained under these conditions in the laboratory for four years (~100 generations of evolution). Thus, the population effectively represents a “laboratory island population”, in which the laboratory environment (and specifically the rearing protocol outlined above) now represents the natural environment in which the population has evolved, and in which most quantitative traits would have had ample time to reach their new evolutionary optima [53]. Admittedly, this laboratory population is much simpler than a natural population, and lacks the multifaceted stresses that natural populations will face. But, it serves as an excellent platform on which to add and assess the effects of multiple stresses, enabling us to assess the transgenerational consequences of dual sleep and pathogen challenges, in the context of the environment in which this population has actually adapted over tens of generations. Recent work on this laboratory population has confirmed abundant levels of quantitative genetic variance for life history trait expression, and strong signatures of transgenerational inheritance mediated by sexual conflict [52, 54, 55].

Prior to the experiment in June 2014, a replicate copy of this population was collected, and the population size expanded to 60 vials per generation, maintained using the same protocols and conditions as described above.

### Experimental details

#### Pathogen treatment

To manipulate maternal and offspring perception of the local pathogenic environment, we challenged flies with a mix of heat-inactivated bacteria composed of the Gram-positive *Micrococcus luteus* and the Gram-negative *Escherichia coli*. There are two main benefits of challenging flies with heat-killed bacteria. Firstly, it allows us to detect the effects a primary immune challenge, whilst excluding the confounding negative effects associated with invading, replicating pathogens [20, 56]. Secondly, and crucially, it ensures that transgenerational effects on offspring fitness are not simply artefacts of the transmission of a live bacterial infection from mother to offspring [24]. Moreover, the rationale behind administering a mix of Gram-positive and Gram-negative bacteria was grounded in the fact that they activate different immune pathways; Gram-positive bacteria primarily stimulate the *Toll* pathway, and Gram-negative bacteria primarily stimulate the *Imd* pathway [38, 57]. Both these pathways are used in the defense against bacteria and fungi, by regulating the production of antimicrobial peptides (AMPs). By triggering both pathways, we speculate that the perceived immune insult may be enhanced, which might possibly generate a stronger response (by additive effects, or by interactive crosstalk) and cause a longer lasting cost in the flies [38, 57, 58].

Virgin females (*N* = 210) were collected from the experimental population, and transferred to vials of 9–14 individuals per vial (x̄ = 11.40 ± SE 0.18), with *ad libitum* access to live yeast provided on the surface of the potato-dextrose-agar-yeast substrate. At three days of adult age, these females were assigned to a maternal pathogen treatment, which consisted of two levels: an injection of heat-killed bacteria or a procedural control (using the nano-injector “Nanoject”, Drummond Scientific Company, Broomall, PA, USA). Half of the females were assigned to the control, and received a microinjection of 41.1 nl of phosphate-buffered saline (PBS, Sigma Aldrich table P4417, pH 7.4) into their abdomens. The other half received the pathogen treatment, which consisted of a cocktail of equal volume of a Gram-positive bacteria (*M. luteus strain,* A204, OD600 = 0.1, corresponding to ~ 1.1 x 10^6^ CFU per fly) and a Gram-negative bacteria (*E. coli,* strain K12, OD600 = 1.0, corresponding to ~ 27.5 x 10^6^ CFU per fly)*,* both provided as heat-killed (verified by colony growth test, supplied by Micromon, Monash University, Australia), and diluted in PBS.

While Drosophila may encounter both *E. coli* and *M. luteus* in the wild [59], it is unclear to what extent these bacterial species may influence *Drosophila* fitness. In fact, little is still known about which species of bacteria that *D. melanogaster* are most likely to come across in their natural environment, and one of the few studies to address host-pathogen associations in wild populations have found it to be both diverse and varied across populations (looking at *D. melanogaster* from the East-coast in the USA) [60]. In contrast, another study exploring the pathogenic species composition across *Drosophila* populations and species throughout the world, found that it was made up of four predominant bacterial families. However, similar to the study by Corby-Harris and Promislow (2008), the sampling distribution in this study was also limited, and while sampling 14 wild *Drosophila* species and two closely related species, only three were in fact *D. melanogaster* populations – all of which, again, were from the USA [59]. Thus, the host-pathogen constellations across species and regions are still largely unknown. Therefore, rather than exactly attempting to mimic natural (bacterial) conditions, we chose to use pathogen species which have proven effects on *Drosophila* immune function and fitness; both bacteria have been shown to have the capacity to induce an immune response in adult *D. melanogaster* (in their live or heat-killed form) [38, 39, 57, 61], and both are capable of influencing reproductive success [19, 39].

The concentration of *E. coli* and *M. luteus* used in the pathogen treatment was based on previous work [19], which had adopted a pilot experiment to test for the effect of different bacterial concentrations on the reproductive success of male and female flies (Additional file 1: Figure S1). Specifically, the final concentration used per pathogen were chosen because they had the largest, and most similar, effects on depressing reproductive success of females.

Finally, the injected volumes were based on established protocols, which has recently also been verified to be within the optimal range for bacterial injections in *Drosophila* [62]. Following the injection, flies were returned to fresh vials, in the absence of live yeast.

#### Sleep deprivation treatment

During the night (starting midnight) following the injections (pathogen treatment *vs*. control), half of the females of each pathogen treatment level were subjected to a sleep deprivation treatment, and the other half to a control. Specifically, the vials of flies assigned to the sleep deprivation were placed on a shaker (Biosan Multi RS-60 rotator, Biosan Medical-Biological Research & Technologies, Riga, Latvia), which was programmed to a cycle starting with a set speed of 3 RPM for 4 min, followed by a 12 s rotation at an angle between -60 and +60°, and finishing with 1° vibrations lasting 1 s. This cycle was repeated continuously for 8 h, at 25 °C in the complete dark (until 8 am). The control females were exposed to the same environment (25 °C, dark), but were not placed on the shakers and hence were not deprived of sleep. The sleep deprivation protocol is based on well-established methods for *Drosophila*, in which sleep is defined as any bout of inactivity that lasts for longer than five continuous minutes [63, 64]. The final protocol adopted in our study was optimized via a pilot study, which demonstrated that the treatment was sufficient to induce sleep-deprivation (Additional file 1: Table S1, Figure S2-3). Briefly, flies subjected to this treatment compensated by sleeping more in the first four hours of daylight, compared with flies subjected to the control (Additional file 1: Table S1. Figure S2). Moreover, flies compensated by sleeping for a substantially longer period of time following the application of the sleep deprivation treatment during night-time hours (i.e. when flies would generally rest) compared to when the sleep-deprivation treatment was applied during daylight hours (Additional file 1: Table S1, Figure S3). Furthermore, the large difference in sleep compensation when the treatment was applied during night-time relative to day-time hours verifies that the response is not merely reflecting a general response to mechanical stress (in which case the flies should compensate equally much regardless of time of day) [64]. Hence, taken together, the results from our sleep treatment pilot study suggest that the sleep deprivation protocol adopted in here was sufficient to disrupt sleep patterns of the flies (more details in the Additional file 1: “Supplementary methods”).

#### Production of focal offspring, offspring pathogen treatments and reproductive assays

On the day following sleep-deprivation (24 h post-injection), an equal number of four days old males from the lab population were added to vials containing virgin females of a sex ratio of 1:1. Flies were allowed to mate for 4 h, after which females were transferred to individual vials for 22 h, to enable ovipositioning. These vials were supplemented with exactly 5 μl of a live yeast slurry (which consisted of 1.2 g yeast dissolved in 10 ml purified water). At the completion of these 22 h, the eggs of each vial were then carefully transferred into fresh vials, containing 6 ml of food substrate, at a density of approximately 25 eggs per vial (x̄ = 22.27 ± SE 0.19, minimum 12 eggs), and females were discarded.

**Fig. 1:**
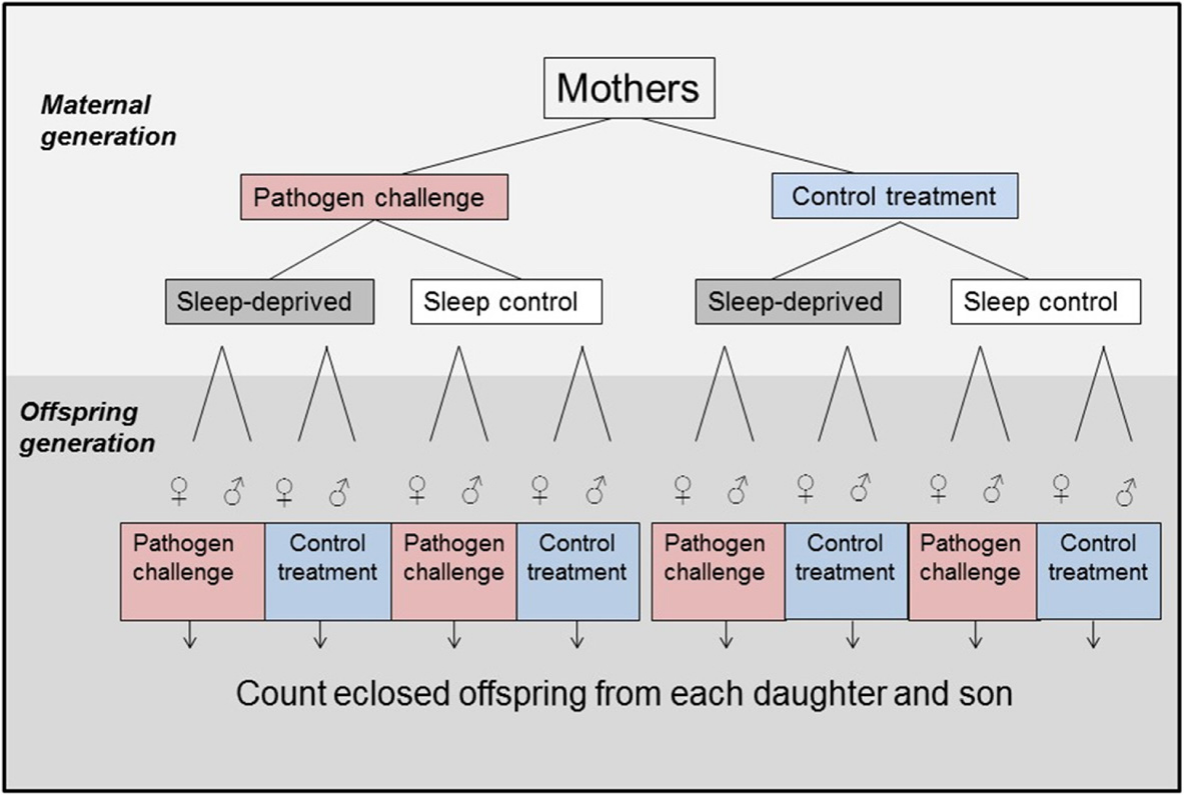
Experimental design. Mothers were first assigned to a pathogen treatment (pathogen-challenged or control) and then allocated to one of two groups, that were either exposed to a night of sleep-deprivation or undisrupted sleep. Two daughters and two sons from of each of these mothers were then exposed to the pathogen treatment (one of each to the challenge and one of each to the control), prior to an assay of their reproductive performance

Nine days later, approximately 30 daughters (x̄ = 29 ± SE 0.4) and 30 sons (x̄ = 34 ± SE 1.7) were collected as virgins from each combination of maternal pathogen and sleep deprivation treatments across each experimental block and placed into three sex-specific vials (x̄ _females_ = 3.3 ± SE 0.3, x̄ _males_ = 3.3 ± SE 0.3) per experimental block. Once these offspring had reached three days of adult age, males and females were assigned to the same pathogen treatment protocol (cocktail or control) as had been applied to the maternal generation, in all possible maternal sleep × pathogen treatment combinations (Fig. 1). In total, approximately 200 focal offspring per maternal sleep – and pathogen treatment combination were injected and then placed in individual, non-yeasted vials for recovery. A four day old virgin fly of the opposite sex was added to each of the vials of focal offspring for 4 h, to enable mating. Following mating, the male flies of each vial were discarded (i.e. tester males in the female assay, and the focal males in the male assay).

A previous study on this population has demonstrated that reproductive output across the first 4 days of ovipositing (starting at age four to five) is tightly correlated with output across the first 10 days [19]. Thus, the females of each vial were retained and allowed to oviposit for the next 96 h, with each female transferred to a new vial every 24 h. All eclosing offspring of these females (the grand-offspring of the treated mothers) were counted 13 days later. Focal female reproductive success was therefore the total number of offspring produced across a 4-day ovipositioning window following a 4 h mating opportunity. Similarly, focal male reproductive success was the total number of offspring produced per male, after a 4 h mating opportunity with a solitary tester female, who was then provided with a 4-day ovipositioning window.

The experiment was repeated over seven full blocks, all of which included all possible treatment and sexes. This generated a final sample of 798 focal male offspring (i.e. the sons of the treated mothers) and 813 focal female offspring (i.e. the daughters), who were each screened for their reproductive success. Note that the initial sample size was larger (854 for females, 848 for males), but was reduced due to mortality (*N* = 57) or flies escaping (*N* = 36) throughout the experiment. We also collected offspring mortality data post-injections, for the 48 period immediately following offspring injections, and the 24 h period immediately following the maternal injections. This data showed that mortality 24 h post-injection did not differ between the pathogen treatment and the control treatment (Dead: N_PBS_ = 31, N_Bac_ = 36, N_total_ = 1031, χ^2^ = 0.37, *p* = 0.54). Likewise, there were no differences in offspring mortality in the 48 h period post-injection for flies subjected to the pathogen challenge relative to the control (*Dead females*: N_PBS_ = 5, N_Bac_ = 0, total females including escapees = 854; *Dead males*: N_PBS_ = 15, N_Bac_ = 11, χ^2^ = 0.62, *p* = 0.56, total males including escapees = 848). There were also no differences in post-injection mortality among the different treatments in female offspring mortality when looking across the entire four day period of ovipositing (i.e., over a 96 h period post-injections, Female deaths: N_PBS_ = 12, N_Bac_ = 14, χ^2^ = 0.15, *p* = 0.85).

#### Statistical analysis

All data were analysed using mixed models in R v. 3.1.1 [65]. The response variable was offspring reproductive success (i.e. number of grand-offspring to sleep-treated × pathogen-treated mothers), and fixed factors were maternal sleep treatment (deprived or control), maternal pathogen treatment (heat-killed bacteria mix or control PBS), and offspring pathogen treatment (heat-killed bacteria mix or control PBS). We also fitted random effects that fully accounted for the structure of the data, i.e. experimental block, vial identity, and maternal identity. The reproductive success of male and female offspring were analysed in separate models, because the assayed trait was not directly comparable across sexes. The female trait is a gauge of reproductive fecundity over four days in early life following a 4 h mating opportunity. The male trait is a gauge of the ability of a male to mate with a female over a 4 h mating window, and measures his subsequent fertility resulting from that mating.

The models were overdispersed, and also zero-inflated (as initially indicated by visual inspection of frequency distribution). We confirmed zero-inflation by 1) comparing all possible models in a fixed framework (i.e. block entered as a factor), which allowed us to apply a Vuong’s test [66] to model assessment, and 2) using a full mixed model, then simulating 95 % confidence intervals (C.I.) around the number of 0 values expected from a true Poisson model corrected for overdispersion. Based on the outcome of these tests, we applied a zero-inflated model, fitted with a negative binomial distribution (to relax the assumption of equal means and variance; NB1 fit, in which variance is calculated as *Øμ*). This was done with the package glmmADMB [67], which utilises a model that allows the zeros to be a mix of structural and sampling data points [68]. In our case, some of the zeros may have been generated because the females did not mate, and others may have arisen from females that had mated but that were infertile themselves, or that mated with infertile males. Both models were type II, and fixed effects were estimated using maximum likelihood.

All interactions up to second order were tested. The effect of higher order interactions were assessed using log-likelihood ratio tests, comparing model deviance between the full model and the reduced model initially, and thereafter, between the progressively reduced models. Hence, model reduction was conducted by removing the least significant interactions (*p* > 0.05) one at a time in a stepwise manner. We present the final model, in which all non-significant interactions has been dropped (Table 1). We also confirmed that the final reduced model had a higher level of empirical support by comparing AIC values between both the null and the full model (AIC difference >2). Raw data has been deposited in Dryad [69].

**Table 1:** Effect of pathogen- and sleep treatments on (a) female and (b) male offspring reproductive performance

*a) Fixed effects*	*df*	*LRT*	*Pr (>χ2)*
Maternal sleep treatment	1	2.78	0.0955
Maternal pathogen treatment	1	0.00	1.0000
Offspring pathogen treatment	1	0.12	0.7290
**Maternal pathogen treatment × Offspring pathogen treatment**	**1**	**5.54**	**0.0186**
*Random effects*	*Variance*		
Parental vial (block)	0.0066		
Maternal identity (Parental vial (block))	0.0192		
*b) Fixed effects*	*df*	*LRT*	*Pr (>χ2)*
Maternal sleep treatment	1	0.02	0.8875
Maternal pathogen treatment	1	0.70	0.4028
Offspring pathogen treatment	1	0.34	0.5598
*Random effects*	*Variance*		
Parental vial (block)	0.0048		
Maternal identity (Parental vial (block))	0.0418		

Log-likelihood ratios (LRT) and their associated *p*-values were generated by comparing the full and reduced models in a stepwise manner, by sequentially removing non-significant terms. Final fit was confirmed by comparing AIC values between the null, reduced, and the full model (AIC > 2). Displayed are final models (significant effects emboldened)

## Results

**Fig. 2:**
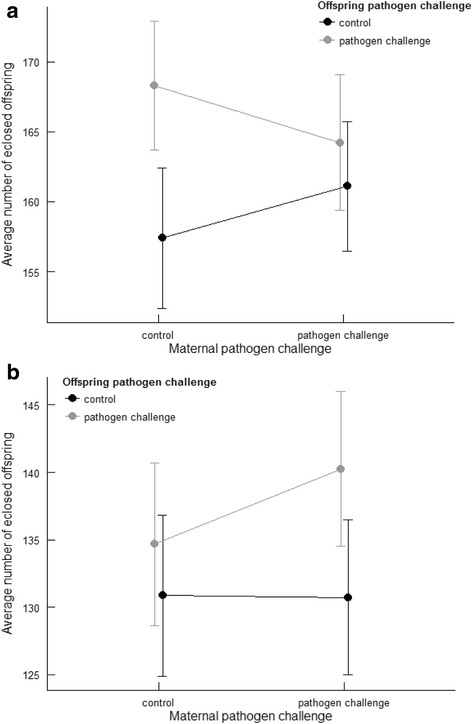
Total number of eclosing offspring (raw means ± SE) produced by **a**) pathogen- and control-treated female offspring produced by pathogen- or control-treated mothers, and **b**) pathogen- and control-treated male offspring produced by pathogen- or control-treated mothers

Reproductive success of the focal female offspring was influenced by an interaction between the maternal and offspring pathogen treatments (Table 1). Female offspring exhibited lower reproductive success when both they, and their mothers, had been administered the control treatment, than when they had received the pathogen treatment but their mothers had received the control (Fig. 2a). The maternal sleep deprivation treatment had no effect on reproductive success of females across generations (Table 1a).

In contrast, male offspring reproductive success was not influenced by the maternal pathogen challenge, maternal sleep treatment, or by the offspring pathogen challenge, nor by any of the interactions between these factors (Table 1b, Fig. 2b).

## Discussion

We screened for pathogen-mediated adaptive transgenerational plasticity in a population of *D. melanogaster*, and investigated whether such effects were modified by an additional maternal stress administered via sleep deprivation. We found that transgenerational interactions, tied to the maternal and offspring pathogen treatments, influenced the reproductive success of female, but not male, offspring. The patterns observed, however, were not consistent with those predicted under an adaptive scenario, whereby pathogen-challenged offspring would be expected to benefit if their mothers had previously faced the same pathogen-challenge and therefore would provide offspring with information on their future (pathogenic) environment. While pathogen-challenged daughters exhibited higher reproductive success than their control counterparts, these patterns were only apparent amongst daughters of control-treated mothers. Thus, pathogen-challenged mothers did not confer any detectable adaptive benefits to their offspring when it came to the reproductive capacity of offspring following an identical pathogen challenge. Rather, the pattern of higher reproductive success amongst pathogen-challenged daughters born to control-treated mothers is possibly more consistent with a process of terminal investment. Furthermore, the reported effects were not in any way altered by the addition of a second maternal stress, imposed by sleep deprivation.

A key prediction of our study was that we would confirm evidence for pathogen-associated transgenerational plasticity, consistent with the results of a previous study in *D. melanogaster* [19]. In that study, both mothers and fathers of two different ages, and their daughters, were subjected to a pathogen-challenge, consisting only of the Gram-positive bacterium *M. luteus*, or a procedural control. While control-treated daughters produced by control-treated mothers exhibited the highest reproductive success, pathogen-challenged daughters performed better if their mothers had likewise received the pathogen-challenge than if their mothers had received the control. However, the results we present here are discordant with those previously observed, with pathogen-challenged female offspring born to control-treated mothers exhibiting higher reproductive success than maternal-offspring combinations in which both parties received the control. A key difference between the present study and the study of Nystrand and Dowling (2014) is that here we used a mix of both heat-killed Gram-negative and Gram-positive bacteria, in an attempt to maximize any potential effects on the immune system by increasing the chances that both the *Imd* and *Toll* pathways would be activated [38]. We note, however, that while there is evidence that these immune pathways can act synergistically [38, 70], this does not necessarily translate to a stronger, additive immune response. Nevertheless, it is conceivable that a more complex pathogen challenge might incur a higher cost to the host. In addition, this complexity could have been further reinforced if the sleep deprivation treatment, indirectly, accentuated the stress to the immune system,by activating the highly-conserved JAK-stat pathway. The JAK-stat signaling pathway, apart from being involved in development and immune function in *Drosophila* (primarily activated by septic injury) [38, 71–73], has recently also been implied to play a role in controlling circadian rhythm by driving rest-activity rhythms [74].

It is also possible that the primary difference between this study and that of Nystrand and Dowling (2014b) is simply a result of dose, given that the double challenge administered here also meant that we injected a much higher concentration of bacteria. Indeed, Nystrand and Dowling (2014a) previously recorded a pronounced dose-dependent effect on reproductive success in Drosophila [56]. Hence, we speculate that the increased effects associated with a double dose of bacteria (i.e. *E. coli* and *M. luteus*) in the current study, might have been sufficient to invoke a response more consistent with a scenario of ‘terminal investment’, and as a result, override any adaptive maternal pathogen-driven fitness effects. The fact that the lowest reproductive success was attributable to control-treated female offspring who were produced by control-treated mothers supports this interpretation. Under a scenario of terminal investment, an individual perceiving an imminent threat to survival should reallocate their resources to current reproduction at the expense of somatic maintenance and survival [75–78].

To this point, we have only discussed the effects of reproductive success in the focal female offspring. We found no pathogen- or sleep-mediated transgenerational effects on the reproductive success of sons. This provides some evidence that the effects of pathogen-mediated transgenerational interactions on offspring fitness are sex-specific, and may disproportionately affect the reproductive success of females. This contention, however, requires further testing. While this is the first study to specifically explore whether pathogen-mediated transgenerational effects adaptively augment the reproductive success of males in *D. melanogaster* (by using a factorial design in which the pathogen treatments of mothers and their offspring were matched or mismatched across offspring sexes), we note that the reproductive assay of male reproductive success adopted here might less accurately reflect male reproductive potential in nature relative to the female assay. We assayed male reproductive success based on a male’s ability to successfully mate with one virgin female, and in the event of doing so, the number of offspring that female went on to produce. Thus, male reproductive success in this assay is based on male pre-copulatory capacity to engage a female in copulation, as well as post-copulatory variation in the quality of the male ejaculate, such as proteomic variation in the constitution of his reproductive proteins, and the number and quality of his sperm transferred during copulation [79, 80]. While female reproductive success is limited by the total number of ova she can oviposit over a given period of time (in this assay, 4 d), male reproductive success can increase as a function of the number of females he has access to [76]. Furthermore, male outcomes are likely to change when assayed under conditions of sperm competition with rival males [80] – a scenario that aligns with the natural mating system of the species. Hence, it will be worthwhile to further investigate the potential for adaptive transgenerational effects manifesting in males, using assays that gauge male fertility outcomes under scenarios of multiple matings or reproductive competition.

A key goal of our study was to determine whether any adaptive maternal effects mediated by a non-infectious pathogen challenge would be further modified in response to an additional stressor. We used sleep deprivation as an inducer of stress, because it is known to upregulate a cluster of immune genes involved in anti-inflammatory responses in *D. melanogaster* [41]. In fact, recent work has established that many of the physiological responses and genes involved in sleep, stress and immunity are related or the same [41, 47, 49, 81–87]. We found no direct transgenerational effects of sleep deprivation on the reproductive success of offspring, and nor did sleep deprivation interact with the maternal or offspring pathogen treatment. We note that our maternal sleep deprivation treatment was applied on the night immediately prior to the mothers reproducing. Thus, it did not represent a chronic sleep stress treatment over many nights, but rather a short-term but intense bout of sleep disruption. We also note that the treated individuals in our study had access to *ad libitum* food, which introduces the potential for compensatory behavior by the flies to offset the effects of the sleep deprivation. In particular, dietary conditions have been shown to affect the outcome of sleep-deprivation in experiments in both humans [88, 89] and rats [90].

Finally, the reported effects on reproductive success are unlikely to be attributable to the effects of selection leading to differential mortality among either the pathogen-treated mothers or pathogen-treated daughters relative to the control-treated counterparts. Mortality levels following the pathogen treatment were invariably low, and no higher amongst mothers or offspring subjected to the pathogen challenge relative to the controls. Thus, our results plausibly reflect true transgenerational interactions tied to maternal effects.

## Conclusions

We documented transgenerational interactions, involving a maternal and an offspring challenge with a non-infectious pathogen, on reproductive success. The observed effects were detected in daughters only, and were not influenced by an additional stress of sleep deprivation in the maternal generation. Furthermore, the transgenerational interactions were not consistent with an interpretation of pathogen-mediated adaptive plasticity, but do provide some preliminary support for a response mediated via terminal investment. Indeed, when considering that this study used a double challenge (whether caused by a more complex response induced by stimulating both main immune pathways, or the result of a simple dose-effect introduced by the administration of higher concentrations of bacteria), relative to a previous study that utilized only a Gram-negative bacterium, this raises the possibility that there might well be a switch-point above which terminal investment effects override those of any potential pathogen-mediated adaptive transgenerational effects. These are research avenues that are amenable to further experimental enquiry, using experimental designs that incorporate dose-dependence and multiple pathogens, of varying perceived pathogenicity (heat-killed and live). Moreover, further exploration of the mechanisms that underpin the pathogen-mediated transgenerational interactions presented here would be valuable, to disentangle whether the observed effects are mediated by transfer of immune factors, a controversial premise [91–93]; or alternatively via classic maternal effects, either condition-dependent and involving reallocation of maternal resources following a challenge with a pathogen, or via epigenetic effects. Such avenues provide a natural extension of this research and an exciting framework for further study into the regulation of pathogen-mediated transgenerational effects, and how such effects may be altered by context-dependence.

## Additional file

Additional file 1: Table S1. Result of pilot experiment exploring sleep disruptive treatment. **Figure S1 a & b.** Results of pilot experiments testing the effect of different concentrations of two different heat-killed pathogens, including procedural controls (PBS) and naïve controls, on reproductive success. **Figure S2 and S3.** Pilot experiment exploring the average number of minutes slept post pathogen-challenge (Figure S2), and post sleep-treatment during the night vs. the day (Figure S3). **Supplementary methods** - Potential influence of the sleep treatment on maternal mating behaviour, and reproductive success, including concomitant effects on offspring reproductive success. (DOC 187 kb)

## References

[1] Mousseau TA, Fox CW (1998). Maternal Effects As Adaptations.

[2] Badyaev AV, Uller T (2009). Parental effects in ecology and evolution: mechanisms, processes and implications. Philos Trans R Soc Lond B Biol Sci.

[3] Marshall D, Uller T (2007). When is a maternal effect adaptive?. Oikos.

[4] Hasselquist D, Nilsson J-Å (2009). Maternal transfer of antibodies in vertebrates: trans-generational effects on offspring immunity. Proc R Soc Lond B Biol Sci.

[5] Grindstaff JL, Brodie ED, Ketterson ED (2003). Immune function across generations: integrating mechanism and evolutionary process in maternal antibody transmission. Proc R Soc Lond B Biol Sci.

[6] Mousseau TA, Fox CW (1998). The adaptive significance of maternal effects. Trends Ecol Evol.

[7] Wolf JB, Wade MJ (2009). What are maternal effects (and what are they not)?. Philos Trans R Soc Lond Ser B: Biol Sci.

[8] Uller T, Nakagawa S, English S (2013). Weak evidence for anticipatory parental effects in plants and animals. J Evol Biol.

[9] Fox CW, Thakar MS, Mousseau TA (1997). Egg size plasticity in a seed beetle: an adaptive maternal effect. Am Nat.

[10] Marshall DJ (2008). Transgenerational plasticity in the sea: context-dependent maternal effects across the life history. Ecology.

[11] Galloway LF, Etterson JR (2007). Transgenerational Plasticity Is Adaptive in the Wild. Science.

[12] Rossiter M (1996). Incidence and consequences of inherited environmental effects. Annu Rev Ecol Syst.

[13] Bernardo J (1996). Maternal Effects in Animal Ecology. Am Zool.

[14] Räsänen K, Kruuk LEB (2007). Maternal effects and evolution at ecological time-scales. Funct Ecol.

[15] Moret Y (2006). 'Trans-generational immune priming': specific enhancement of the antimicrobial immune response in the mealworm beetle, *Tenebrio molitor*. Proc R Soc Lond Ser B: Biol Sci.

[16] Roth O, Joop G, Eggert H, Hilbert J, Daniel J, Schmid-Hempel P, Kurtz J (2010). Paternally derived immune priming for offspring in the red flour beetle, *Tribolium castaneum*. J Anim Ecol.

[17] Hernández López J, Schuehly W, Crailsheim K, Riessberger-Gallé U (2014). Trans-generational immune priming in honeybees. Proc R Soc Lond B Biol Sci.

[18] Little TJ, O'Connor B, Colegrave N, Watt K, Read AF (2003). Maternal Transfer of Strain-Specific Immunity in an Invertebrate. Curr Biol.

[19] Nystrand M, Dowling DK (2014). Transgenerational interactions involving parental age and immune status affect female reproductive success in *Drosophila melanogaster*. Proc R Soc Lond Ser B: Biol Sci.

[20] Sadd BM, Kleinlogel Y, Schmid-Hempel R, Schmid-Hempel P (2005). Trans-generational immune priming in a social insect. Biol Lett.

[21] Tidbury HJ, Pedersen AB, Boots M (2011). Within and transgenerational immune priming in an insect to a DNA virus. Proc R Soc Lond Ser B: Biol Sci.

[22] Zanchi C, Troussard J-P, Martinaud G, Moreau J, Moret Y (2011). Differential expression and costs between maternally and paternally derived immune priming for offspring in an insect. J Anim Ecol.

[23] Little TJ, Kraaijeveld AR (2004). Ecological and evolutionary implications of immunological priming in invertebrates. Trends Ecol Evol.

[24] Freitak D, Schmidtberg H, Dickel F, Lochnit G, Vogel H, Vilcinskas A (2014). The maternal transfer of bacteria can mediate trans-generational immune priming in insects. Virulence.

[25] Grindstaff JL (2008). Maternal antibodies reduce costs of an immune response during development. J Exp Biol.

[26] Mousseau TA, Dingle H (1991). Maternal Effects in Insect Life Histories. Annu Rev Entomol.

[27] Curley JP, Mashoodh R, Champagne FA (2011). Epigenetics and the origins of paternal effects. Horm Behav.

[28] Jablonka E, Raz G (2009). Transgenerational Epigenetic Inheritance: Prevalence, Mechanisms, and Implications for the Study of Heredity and Evolution. Q Rev Biol.

[29] Salmela H, Amdam GV, Freitak D (2015). Transfer of Immunity from Mother to Offspring Is Mediated via Egg-Yolk Protein Vitellogenin. PLoS Path.

[30] Sadd BM, Schmid-Hempel P (2009). A distinct infection cost associated with trans-generational priming of antibacterial immunity in bumble-bees. Biol Lett.

[31] Prior NH, Washington CN, Housley JM, Hall SR, Duffy MA, Caceres CE (2011). Maternal effects and epidemiological traits in a planktonic host-parasite system. Evol Ecol Res.

[32] Sadd BM, Schmid-Hempel P (2007). Facultative but persistent trans-generational immunity via the mother's eggs in bumblebees. Curr Biol.

[33] Plaistow SJ, Benton TG (2009). The influence of context-dependent maternal effects on population dynamics: an experimental test. Philos Trans R Soc Lond B Biol Sci.

[34] Agrawal AA (2001). Transgenerational consequences of plant responses to herbivory: an adaptive maternal effect?. Am Nat.

[35] Lazzaro BP, Little TJ (2009). Immunity in a variable world. Philos Trans R Soc Lond B Biol Sci.

[36] Triggs AM, Knell RJ (2012). Parental diet has strong transgenerational effects on offspring immunity. Funct Ecol.

[37] Eggert H, Diddens-de Buhr MF, Kurtz J (2015). A temperature shock can lead to trans-generational immune priming in the Red Flour Beetle, *Tribolium castaneum*. Ecol Evol.

[38] Lemaitre B, Hoffmann J (2007). The Host Defense of *Drosophila melanogaster*. Annu Rev Immunol.

[39] Zerofsky M, Harel E, Silverman N, Tatar M (2005). Aging of the innate immune response in *Drosophila melanogaster*. Aging Cell.

[40] Svetec N, Zhao L, Saelao P, Chiu JC, Begun DJ (2015). Evidence that natural selection maintains genetic variation for sleep in *Drosophila melanogaster*. BMC Evol Biol.

[41] Williams J, Sathyanarayanan S, Hendricks J, Sehgal A (2007). Interaction between sleep and the immune response in *Drosophila*: a role for the NFkappaB relish. Sleep.

[42] Koh K, Evans JM, Hendricks JC, Sehgal A (2006). A *Drosophila* model for age-associated changes in sleep:wake cycles. Proc Natl Acad Sci USA.

[43] Bushey D, Cirelli C. From Genetics to Structure to Function: Exploring Sleep in Drosophila. Int Rev Neurobiol. 2011;(99):213–44. 10.1016/B978-0-12-387003-2.00009-4 PMC3172676 21906542

[44] Kuo T-H, Pike D, Beizaeipour Z, Williams J (2010). Sleep triggered by an immune response in *Drosophila* is regulated by the circadian clock and requires the NFkappaB Relish. BMC Neurosci.

[45] Cirelli C (2009). The genetic and molecular regulation of sleep: from fruit flies to humans. Nat Rev Neurosci.

[46] Cirelli C, Tononi G (2008). Is Sleep Essential?. PLoS Biol.

[47] Shaw PJ, Tononi G, Greenspan RJ, Robinson DF (2002). Stress response genes protect against lethal effects of sleep deprivation in *Drosophila*. Nature.

[48] Li X, Yu F, Guo A (2009). Sleep Deprivation Specifically Impairs Short-term Olfactory Memory in Drosophila. Sleep.

[49] Kuo T-H, Williams JA (2014). Increased Sleep Promotes Survival during a Bacterial Infection in *Drosophila*. Sleep.

[50] Cirelli C, LaVaute TM, Tononi G (2005). Sleep and wakefulness modulate gene expression in *Drosophila*. J Neurochem.

[51] Kuo TH, Williams JA (2014). Acute sleep deprivation enhances post-infection sleep and promotes survival during bacterial infection in *Drosophila*. Sleep.

[52] Williams BR, Van Heerwaarden B, Dowling DK, SgrÒ CM (2012). A multivariate test of evolutionary constraints for thermal tolerance in *Drosophila melanogaster*. J Evol Biol.

[53] Rice W, Linder J, Friberg U, Lew T, Morrow E, Stewart A (2005). Inter-locus antagonistic coevolution as an engine of speciation: Assessment with hemiclonal analysis. Proc Natl Acad Sci U S A.

[54] Dowling DK, Williams BR, Garcia-Gonzalez F (2014). Maternal sexual interactions affect offspring survival and ageing. J Evol Biol.

[55] Garcia-Gonzalez F, Dowling DK: Transgenerational effects of sexual interactions and sexual conflict: non-sires boost the fecundity of females in the following generation. Biol Lett 2015, 11(3).doi: 10.1098/rsbl.2015.0067. 10.1098/rsbl.2015.0067 PMC4387503 25788486

[56] Nystrand M, Dowling DK (2014). Dose-dependent effects of an immune challenge at both ultimate and proximate levels in *Drosophila melanogaster*. J Evol Biol.

[57] Nehme NT, Quintin J, Cho JH, Lee J, Lafarge M-C, Kocks C, Ferrandon D (2011). Relative Roles of the Cellular and Humoral Responses in the *Drosophila* Host Defense against Three Gram-Positive Bacterial Infections. PLoS One.

[58] McKean K, Yourth C, Lazzaro B, Clark A (2008). The evolutionary costs of immunological maintenance and deployment. BMC Evol Biol.

[59] Chandler JA, Morgan Lang J, Bhatnagar S, Eisen JA, Kopp A (2011). Bacterial Communities of Diverse *Drosophila* Species: Ecological Context of a Host–Microbe Model System. PLoS Genet.

[60] Corby-Harris V, Promislow DEL (2008). Host ecology shapes geographical variation for resistance to bacterial infection in *Drosophila melanogaster*. J Anim Ecol.

[61] Elrod-Erickson M, Mishra S, Schneider D (2000). Interactions between the cellular and humoral immune responses in *Drosophila*. Curr Biol.

[62] Khalil S, Jacobson E, Chambers MC, Lazzaro BP (2015). Systemic Bacterial Infection and Immune Defense Phenotypes in *Drosophila Melanogaster*. J Vis Exp.

[63] Shaw P, Cirelli C, Greenspan R, Tononi G (2000). Correlates of sleep and waking in *Drosophila melanogaster*. Science.

[64] Cirelli C (2003). Searching for sleep mutants of *Drosophila melanogaster*. Bioessays.

[65] R Development Core Team (2012). R: A Language and Environment for Statistical Computing. R Foundation for Statistical Computing.

[66] Vuong QH (1989). Likelihood Ratio Tests for Model Selection and Non-Nested Hypotheses. Econometrica.

[67] Skaug HJ, Fournier DA, Nielsen A, Magnusson A, Bolker BM: Package glmmADMB: Generalized linear mixed models using AD Model Builder. R package version 0.7.3. http://r-forge.r-project.org/projects/glmmadmb/. 2012.

[68] Hu M-C, Pavlicova M, Nunes EV (2011). Zero-inflated and Hurdle Models of Count Data with Extra Zeros: Examples from an HIV-Risk Reduction Intervention Trial. Am J Drug Alcohol Abuse.

[69] Nystrand M, Cassidy EJ, Dowling D (2016). Data from: Transgenerational plasticity following a dual pathogen and stress challenge in fruit flies. Dryad Digital Repository. http://dx..

[70] Valanne S, Wang J-H, Rämet M (2011). The *Drosophila* Toll Signaling Pathway. J Immunol.

[71] Agaisse H, Perrimon N (2004). The roles of JAK/STAT signaling in *Drosophila* immune responses. Immunol Rev.

[72] Arbouzova NI, Zeidler MP (2006). JAK/STAT signalling in *Drosophila*: insights into conserved regulatory and cellular functions. Development.

[73] Wang H, Chen X, He T, Zhou Y, Luo H (2013). Evidence for Tissue-Specific JAK/STAT Target Genes in *Drosophila* Optic Lobe Development. Genetics.

[74] Luo W, Sehgal A (2012). Regulation of circadian behavioral output via a MicroRNA-JAK/STAT circuit. Cell.

[75] Clutton-Brock TH (1984). Reproductive Effort and Terminal Investment in Iteroparous Animals. Am Nat.

[76] Bateman AJ (1948). Intra-sexual selection in *Drosophila*. Heredity (Edinb).

[77] Roff DA. Life History Evolution. Sunderland: Sinauer; 2002

[78] Williams GC (1966). Natural Selection, the Costs of Reproduction, and a Refinement of Lack's Principle. Am Nat.

[79] Simmons LW: Sperm Competition and Its Evolutionary Consequences in the Insects. New Jersey: Princeton University Press; 2001.

[80] Yee WKW, Sutton KL, Dowling DK (2013). In vivo male fertility is affected by naturally occurring mitochondrial haplotypes. Curr Biol.

[81] Meerlo P, Sgoifo A, Suchecki D (2008). Restricted and disrupted sleep: Effects on autonomic function, neuroendocrine stress systems and stress responsivity. Sleep Med Rev.

[82] Harbison ST, Mackay TFC, Anholt RRH (2009). Understanding the neurogenetics of sleep: progress from *Drosophila*. Trends Genet.

[83] Zimmerman J, Naidoo N, Raizen D, Pack A (2008). Conservation of sleep: insights from non-mammalian model systems. Trends Neurosci.

[84] Adamo SA (2010). Why should an immune response activate the stress response? Insights from the insects (the cricket *Gryllus texensis*). Brain, Behav Immun.

[85] Maier SF (2003). Bi-directional immune–brain communication: Implications for understanding stress, pain, and cognition. Brain Behav Immun.

[86] Elenkov IJ, Chrousos GP (2006). Stress System – Organization, Physiology and Immunoregulation. Neuroimmunomodulation.

[87] Badyaev AV (2005). Stress-induced variation in evolution: from behavioural plasticity to genetic assimilation. Proc R Soc Lond Ser B: Biol Sci.

[88] Fang Z, Spaeth AM, Ma N, Zhu S, Hu S, Goel N, Detre JA, Dinges DF, Rao H (2015). Altered salience network connectivity predicts macronutrient intake after sleep deprivation. Sci Rep.

[89] Markwald RR, Melanson EL, Smith MR, Higgins J, Perreault L, Eckel RH, Wright KP (2013). Impact of insufficient sleep on total daily energy expenditure, food intake, and weight gain. Proc Natl Acad Sci USA.

[90] Thimgan MS, Gottschalk L, Toedebusch C, McLeland J, Rechtschaffen A, Gilliland-Roberts M, Duntley SP, Shaw PJ (2013). Cross-Translational Studies in Human and *Drosophila* Identify Markers of Sleep Loss. PLoS One.

[91] Hauton C, Smith VJ (2007). Adaptive immunity in invertebrates: A straw house without a mechanistic foundation. Bioessays.

[92] Little TJ, Colegrave N, Sadd BM, Schmid-Hempel P (2008). Studying immunity at the whole organism level. Bioessays.

[93] Rowley AF, Powell A (2007). Invertebrate Immune Systems–Specific, Quasi-Specific, or Nonspecific?. J Immunol.

