# Supplementary material for: Transgenerational plasticity following a dual pathogen and stress challenge in fruit flies
Source: BMC Evol Biol. 2016 Aug 27;16(1):171. doi: 10.1186/s12862-016-0737-6 (PMC5002108; doi:10.1186/s12862-016-0737-6)
Supplement: Additional file 1: Table S1. — Result of pilot experiment exploring sleep disruptive treatment. Figure S1 a & b. Results of pilot experiments testing the effect of different concentrations of two different heat-killed pathogens, including procedural controls (PBS) and naïve controls, on reproductive success. Figure S2 and S3. Pilot experiment exploring the average number of minutes slept post pathogen-challenge (Figure S2), and post sleep-treatment during the night vs. the day (Figure S3). Supplementary methods - Potential influence of the sleep treatment on maternal mating behaviour, and reproductive success, including concomitant effects on offspring reproductive success. (DOC 187 kb) [file 12862_2016_737_MOESM1_ESM.doc]

**Transgenerational plasticity following a dual pathogen and stress challenge in fruit flies**

Nystrand, M*, Cassidy, EJ. & Dowling, DK

***School of Biological Sciences, Monash University, Clayton, Victoria, 3800, Australia***

***magdalena.nystrand@monash.edu***

***elizabethjanecassidy@gmail.com***

***damian.dowling@monash.edu***

**** Correspondence:***

*Dr Magdalena Nystrand, School of Biological Sciences, Monash University,*

*Clayton, Victoria, 3800, Australia*

phone: +61-(0)3-9902 4346, fax: +61-(0)3-9905 5613

email: [magdalena.nystrand@monash.edu](mailto:magdalena.nystrand@monash.edu)

**Table of contents**

1. **Table S1.** Result of pilot experiment exploring sleep disruptive treatment.
2. **Figure S1 a & b.** Results of pilot experiments testing the effect of different concentrations of two different heat-killed pathogens, including procedural controls (PBS) and naïve controls, on reproductive success.

**Figure S2 and S3.** Pilot experiment exploring the average number of minutes slept post pathogen-challenge (Fig. S2), and post sleep-treatment during the night vs. the day (Fig. S3).

1. **Supplementary methods** - *Potential influence of the sleep treatment on maternal mating behaviour, and reproductive success, including concomitant effects on offspring* *reproductive success.*
2. **References**

**1. Tables**

**Table S1.** Result of pilot experiment (square root transformed response variable, Gaussian distribution) investigating the effect of the sleep deprivation treatment versus the sleep controls. Identical sleep deprivation treatments were conducted across 10 blocks (n = 5 night time, n = 5 day time). Response variable is the average number of minutes slept (i.e. with sleep defined as a bout of inactivity ≥ 5 minutes) in the four hours following the sleep deprivation treatment or control treatment. Significant effects are emboldened.

| ***Fixed effects*** | ***df*** | ***LRT*** | ***Pr (>χ2)*** |
| --- | --- | --- | --- |
| **Sleep treatment** | **1** | **4.4906** | **0.0341** |
| **Time of day (day or night)** | **1** | **5.9475** | **0.0147** |
| Sleep treatment × Time of day | 1 | 0.4163 | 0.5188 |
| ***Random effects (best model: AIC)*** | ***Full model Variance*** | | |
| Block (Time of day) | 1.4540  12.702 | | |
| Residual |

**2. Figures**

**a)**

**
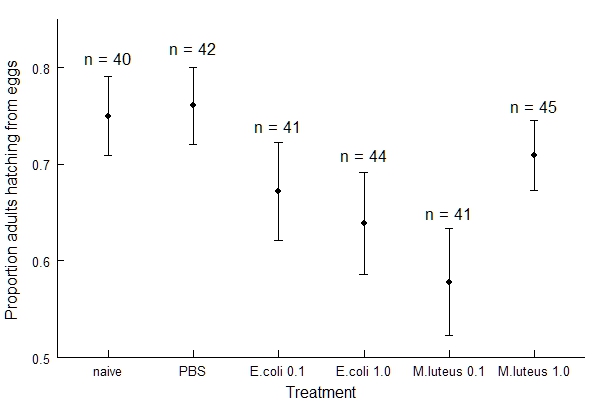
**

**b)**


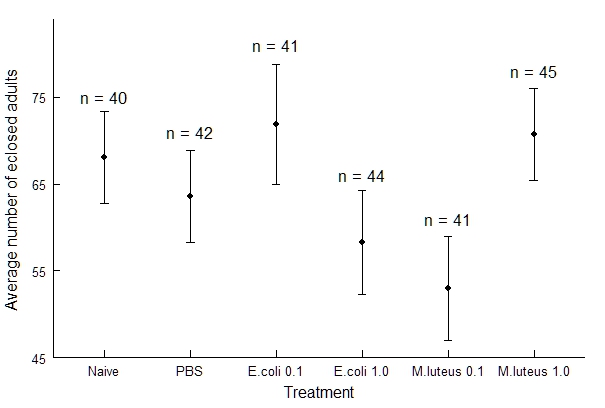


**Figure S1.** Graphs displaying raw mean ± SE of the a) proportion adults hatching out from eggs, and b) average number of eclosed adults per treatment, that eclosed over a period of 4 days after females had been given one of the following treatments: naïve, PBS (procedural control), *E. coli* OD_600_ = 0.1, *E. coli* OD_600_ = 1.0, *M. Luteus* OD_600_ = 0.1, and *M. Luteus* OD_600_ = 1.0. Note that S1a reflects effects on density-controlled reproductive success (i.e. it displays number of adults in relation to number of eggs laid per female), which most mimics the conditions experienced by the mothers in the experiment presented in the main text whereby the clutch size was standardized to 25 eggs per vial. In contrast, S1b is not density controlled. Statistical output for S1a: GLM, quasibinomial error, dev = 303.11, p = 0.041, and for S1b: glmmADMB, negative binomial error (NB1), dev = 13.86, p < 0.020.


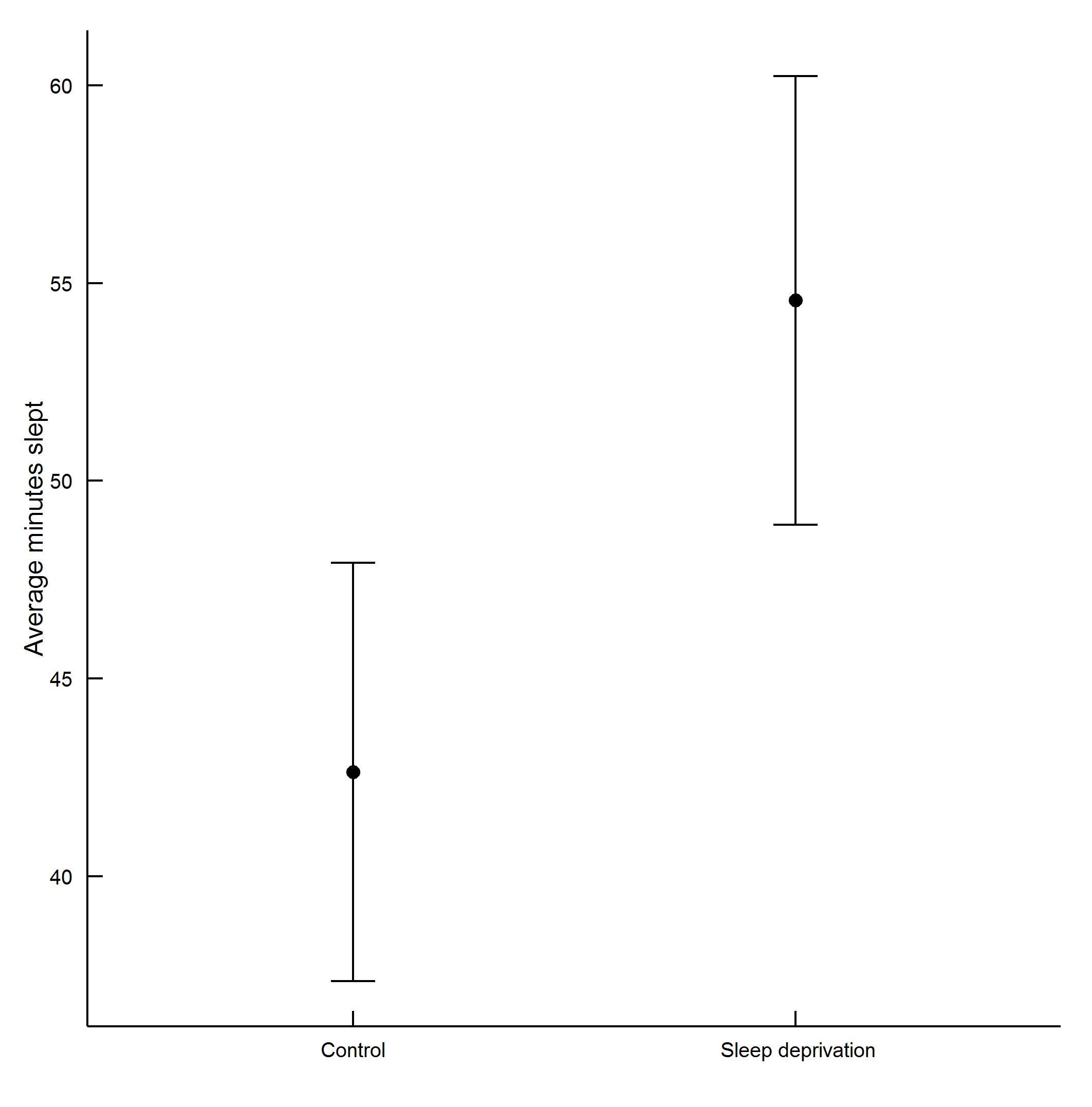


**Figure S2.** Mean ± SE number of minutes slept by focal flies (i.e. bouts of inactivity ≥ 5 minutes) in the 4 h following exposure to sleep deprivation relative to flies exposed to the control.


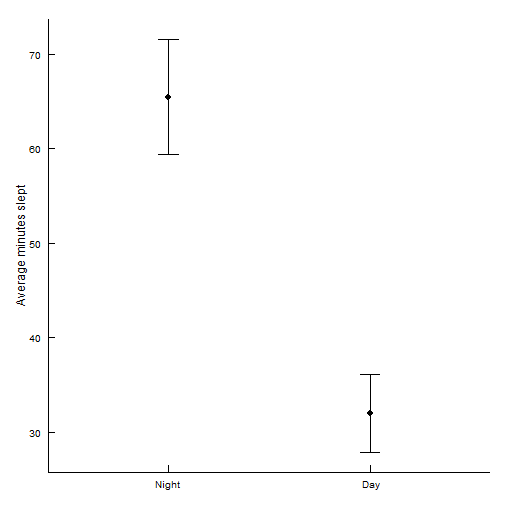


**Figure S3.** The mean number ± SE of minutes slept by focal flies (bouts of inactivity ≥ 5 minutes) following the sleep treatment; when the sleep treatment was applied during the night versus during the day.

**3. Supplementary methods**

*Potential influence of the sleep treatment on maternal mating behaviour, and reproductive success, including concomitant effects on offspring* *reproductive success*

It is possible that the sleep deprivation treatment directly influenced maternal mating behaviour. Although we did not specifically monitor mating behaviour, nor did we specifically record maternal reproductive output (i.e. we selected four offspring from each mother, regardless of how many offspring she had produced), we were able to indirectly assess behaviour-induced changes in mating patterns by comparing the number of eggs laid across sleep treatments. This comparison is grounded on the premise that, had there been negative changes in maternal mating behaviour due to influences from the sleep treatment, we would have expected to record more zero reproductive events in the females subjected to the sleep treatment relative to the control (indicative of the sleep-treated females failing to mate), and / or a lowered reproductive success of these sleep-treated females. It is unlikely that the sleep treatment would have resulted in female mating rates that were elevated above 1 mating during the designated exposure to males. This assumption is based on the fact that females in our study only had a 4 hr window of opportunity to mate, and *Drosophila* females are known to have long refractory periods following initial matings – often ranging beyond 24 hr [1-3]. Further support for this argument comes from previous studies showing that *Drosophila* evolve low remating rates in laboratory populations [4], and in many lab populations, females will mate only once within the first couple of hours of exposure to males, prior to entering their refractory period [5]. Hence, given the short mating bouts in our study, it is likely that females would have mated only once, or not at all, and hence we are able to compare these mating rates across the treatment.

We used data from maternal egg laying density recordings from all blocks, in which we had recorded the number of eggs laid per female (one vial per female) in order to cap vial densities to around 25 eggs. Because some females failed to produce 25 eggs within the required amount of time, this data could be used as an indication of some females performing worse than others (i.e. lower average number of eggs, or higher number of zero reproductive output). Our data did not suggest that the sleep treatment influenced the mating behaviour in the mothers because not only was the number of females generating zero eggs similar between treatments (N_control_ = 126 [N_total_ = 506 females], N_sleep-deprived_ = 120 [N_total_ = 500 females], χ^2^ = 0.10, df = 1, p-value = 0.75), but the average number of total eggs laid was also similar (X_Control_ = 16.04, SE = 0.46, N = 506; X_sleep-deprived_ = 16.16, SE =0.46, N = 500, χ^2^ = 0.04, df = 1, p-value = 0.84). Finally, a comparison of egg numbers limited to only those females that did not lay 25 eggs generated similar results: (X_Control_ [N = 294 females] = 9.47 eggs, SE = 0.54; X_sleep-deprived_ [N = 286] = 9.54 eggs, SE =0.54).

In addition, we did not detect any carry-over effects from the maternal sleep treatment on offspring mating behaviour, as indicated by a presence of zero reproductive output (zero values indicate offspring failed to mate, or possibly, were sterile) in the offspring dataset across the Sleep treatment versus Control: (Females: Nsleep = 35, Ncontrol = 22 , χ2 = 2.50, p = 0.11; Males: Nsleep = 76, Ncontrol = 75 , χ2 = 0.00, p = 1.00 ). This trend also held true when focusing only on the first day following bacterial injection, at which time point the offspring can be expected to be most sensitive due to the recent added stress of pathogen exposure (Females: Nsleep= 36, Ncontrol = 22, χ2 = 2.41, p = 0.12; Males: Nsleep= 77, Ncontrol = 75, χ2 = 0.00, p = 1.00).

1. **References**

1. Manning A: **A Sperm Factor Affecting the Receptivity of Drosophila Melanogaster Females**. *Nature* 1962, **194**(4825):252-253.

2. Manning A: **The control of sexual receptivity in female Drosophila**. *Anim Behav* 1967, **15**(2–3):239-250.

3. Scott D: **The timing of the sperm effect on female Drosophila melanogaster receptivity**. *Anim Behav* 1987, **35**(1):142-149.

4. Chapman T, Partridge L: **Female Fitness in Drosophila melanogaster: An Interaction between the Effect of Nutrition and of Encounter Rate with Males**. *Proc R Soc Lond, Ser B: Biol Sci* 1996, **263**(1371):755-759.

5. Holland B, Rice W: **Experimental removal of sexual selection reverses intersexual antagonistic coevolution and removes a reproductive load**. *Proc Natl Acad Sci U S A* 1999, **96**:5083 - 5088.
